# Presence of Fermentable Oligo-, Di-, Monosaccharides, and Polyols (FODMAPs) in commonly eaten foods: extension of a database to indicate dietary FODMAP content and calculation of intake in the general population from food diary data

**DOI:** 10.1186/s40795-020-00374-3

**Published:** 2020-09-18

**Authors:** Therese Liljebo, Stine Störsrud, Anna Andreasson

**Affiliations:** 1 The City Gastrodepartment, Stockholm, Sweden; 2 Department of Internal Medicine and Clinical Nutrition, Institute of Medicine, Sahlgrenska Academy, University of Gothenburg, Gothenburg, Sweden; 3 Stress Research Institute, Stockholm University, SE-109 61 Stockholm, Sweden; 4 Department of Medicine Solna, Karolinska Institutet, Stockholm, Sweden

**Keywords:** FODMAP, General population, Food diary survey, Fructan, Galacto-oligosaccharides, Polyols

## Abstract

**Background:**

FODMAPs (Fermentable Oligo-, Di-, Monosaccharides And Polyols) are known for their health benefits but their fermentation may trigger gastrointestinal symptoms and a low-FODMAP diet is a commonly used intervention for functional gastrointestinal disorders. The use of direct measures of FODMAP is labor intensive and expensive and to facilitate the assessment of FODMAP intake in research and clinical work, a nutritional content database with good quality estimates on FODMAP values is needed. Further, the average intake of FODMAP in a general population would be a useful reference and knowledge of the most commonly eaten foods containing FODMAPs would facilitate clinical work utilizing FODMAP diet interventions.

**Methods:**

A nutritional content database was extended with published FODMAP content data. The database was used to calculate FODMAP intake from four-day food diaries from 117 individuals from the general population in Sweden and the most common food items containing FODMAPs were identified.

**Results:**

FODMAP content for 1060 food items was added to the database resulting in 1805 listed FODMAP values. Mean intake of total FODMAP in the diaries was 19 g (fructose: 15.2 g; fructan: 3.5 g; lactose: 14.1 g; galacto-oligosaccharides (GOS) 0.43 g and polyols 1.3 g per day). Overall the most common eaten food items containing FODMAPs were rye and wheat based foods.

**Conclusion:**

Intake of FODMAPs as calculated using the extended database were in line with previous studies supporting its use of the database in both research and clinical interventions. The lists of the most commonly eaten FODMAP food items are provided and may be used to facilitate FODMAP diet interventions.

## Background

FODMAPs (Fermentable Oligo- Di- Monosaccharides and Polyols) include the sugar monomer fructose and the fermentable short chain carbohydrates (SCC) including fructans (inulin and fructo- oligosaccharides), lactose, galacto-oligosaccharides (GOS) and polyols [1]. Fructans and GOS are known for their prebiotic effects [2]. After reaching the colon intact they undergo bacterial fermentation and formation of short chain fatty acids as butyrate, propionic acid and acetate [3], and health benefits of FODMAPs include stimulated bifidobacteria growth [4], increased calcium absorption and improved appetite regulation [5]. In spite of the health benefits, the intestinal fermentation of FODMAPs may trigger gastrointestinal symptoms in patients with irritable bowel syndrome (IBS) and inflammatory bowel disease (IBD) and treatment with a low FODMAP diet seems a promising intervention for these conditions [6].

FODMAPs are present in a large variety of foods. Fructose is present in fruit juices and honey. Lactose is present in dairy products, fructans in artichokes, garlic and onions, and GOS in legumes. Polyols are present as sweeteners in chewing gums, avocado, mushrooms, apples and pear [1]. Since fructose is only co-absorbed with glucose, fructose in excess of glucose [7] is counted towards the FODMAP total. High performance liquid chromatography (HPLC) with evaporative light scattering detection (ELSD), gas-chromatographic (GC) and enzymatic analyses are the most commonly used methods for analyzing FODMAPs, and the method depend on the specific FODMAP to be analyzed. There are several studies from around the world that have investigated the FODMAP content in food items, including Australia [8–10], United States [11, 12], and Europe [13, 14].

Country specific information about the habitual intake, and typically sources of FODMAPs are needed to facilitate nutrition intervention and therapy that entails changing the intake of FODMAPs. The food database from the Swedish National Food Agency does not contain any values of FODMAP content, except from lactose and fructose [15, 16]. Furthermore, there are no previous reports of the most commonly eaten food items containing FODMAPs in Sweden or elsewhere. Thus, data on intake of FODMAPs and their main sources in the general population are lacking.

The primary aim of the present study was to systematically add published values on FODMAPs from peer-reviewed literature and reports to a nutrition calculation software program Dietist XP (Kostdata.se). The secondary aim was to calculate the intake of fructose, fructan, lactose, GOS and polyols in a sample of healthy individuals from the general population in Sweden and compare to previous research using direct analyses of FODMAPs. In addition, we listed the most commonly eaten food items containing each type of FODMAPs.

## Methods

### Expanding the food database with values of FODMAP content

**Fig. 1:**
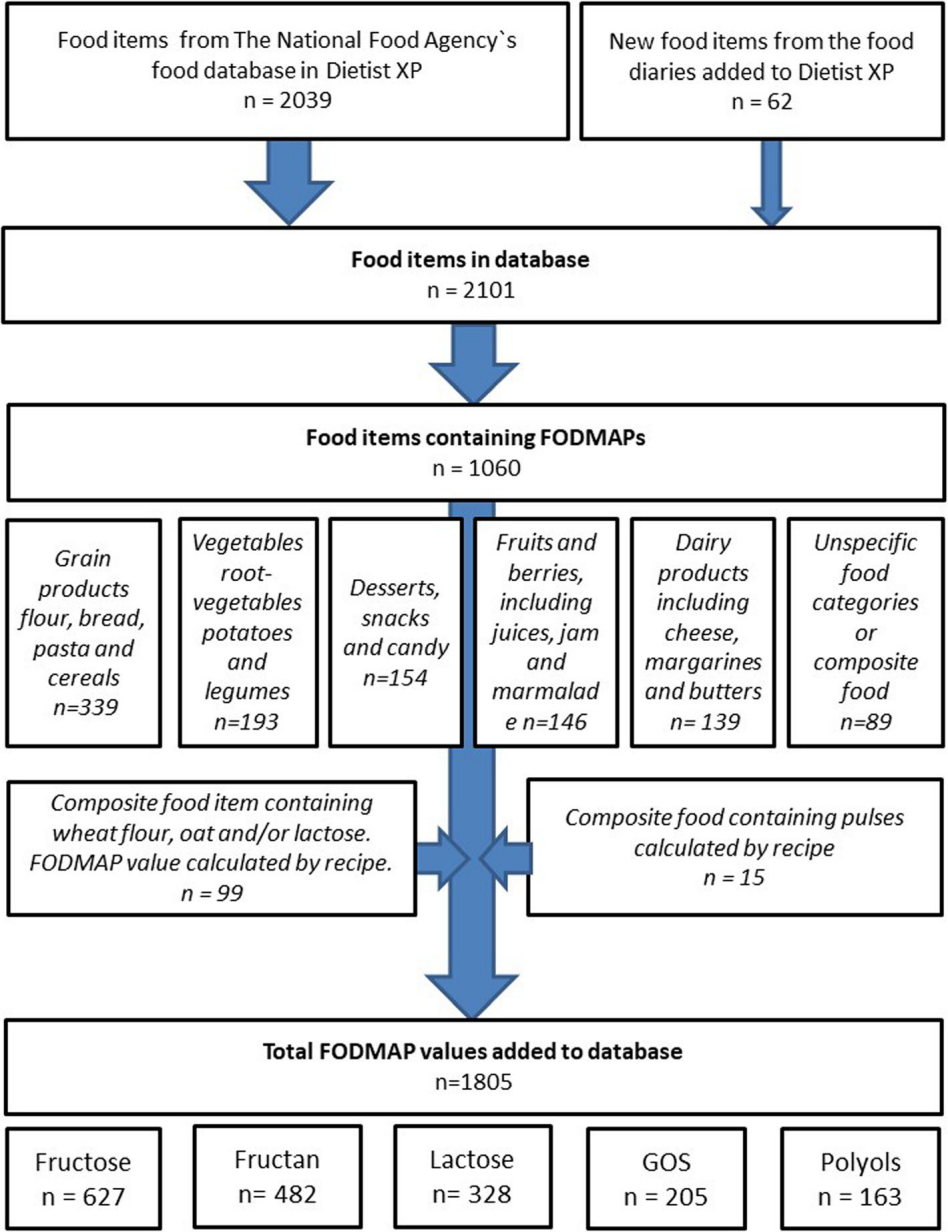
Flowchart describing the process of assigning values to the database. FODMAP=Fermentable-oligo-di-monosaccharides and polyols, GOS = galacto–oligosaccharides

The process to expand the food database with values of FODMAPs is illustrated in Fig. 1. First the five groups of FODMAPs; fructose, fructan, lactose, GOS and polyols, were added to the list of nutrients within the software program Dietist XP (version 3:2, Kostdata.se). Dietist XP is a nutrition calculation software program widely used in Sweden and is based on the official food database from the Swedish National Food Agency (2012-03-19). Second, all the existing 2039 food item or dishes in Dietist XP was manually checked against reports containing FODMAP-data from Swedish National Food Agency published in year 2007 to 2014 and published peer-reviewed papers. In addition, food items missing from the database but reported in the food diaries (see below) were added (Table 1). Non-carbohydrate foods such as meat, fish and egg were not included in the database as they consequently contain no FODMAPs. Baby food was not included as the database is intended for assessment of FODMAP intake in adults. Swedish sources were used when possible.

**Table 1:** Main peer- reviewed papers used as source for FODMAP values

References	Analyzed food item/ product/ raw material	Analyzed value added in database	Food sampling/biological variation	Analytical method
[16] National Swedish Food Agency 2007–2013	One hundred and nineteen vegetables, cereal products and other food items	Fructose and lactose	Purchased in grocery chains, small food stores, and vegetable retailers and from casual trading area. • If possible a minimum of ten samples of each food item	• Gas- chromatographic method [17]. • Result presented in fresh weight g/100 g food item
[9] Muir et al.2007	Sixty vegetables and 43 fruits	Free fructose and fructan	Approximately 500 g (edible weight) of each food item from respectively five grocery stores and five green grocers, Australia, Melbourne	• Analyses made of pooled samples • Enzymatic analysis and spectrophotometry [18] • Triplicate analysis • Result presented in g/ 100 g “as eaten” in fresh weight
[10] Muir et al.2009	Forty-five vegetables and 41 fruits	Fructose, fructan	Approximately 500 g (edible weight) of each food item from each of five grocery stores and five green grocers, Australia, Melbourne	• Analyses made of pooled samples •HPLC with ELSD •Triplicate analysis •Result presented in g/ 100 g “as eaten” in fresh weight
[8] Biesiekierski et al. 2011	Fifty- five grains and cereals	Fructose, FOS (nystose, kestose). Total fructan, lactose, GOS (raffinose, stachyose) sugar polyols (sorbitol, mannitol)	Approximately 500 g (edible weight) of each food item from Supermarkets, market- places and health stores in Melbourne, Australia, One to 9 products/ brands of each food item, 500 g of each product edible weight	• Analyses made of pooled samples • HPLC • Total fructan via enzymatic analyses [19] • Result presented in g/ 100 g “as eaten” in fresh weight.
[20] Whelan et al.2011	Nine categories of bread	Fructan	Five brands of each bread category and 500 g of each category of bread from Supermarkets was pooled together to 2500 g	• Analyses made of pooled samples • Fructan via enzymatic and spectrophotometry method [18] Triplicate bread samples were extracted and analyzed in duplicates. • Result presented as content g/100 g fresh weight (‘as consumed’)
[13] Andersson et al.2009	Rye kernels and five kind of whole grain rye soft- and crispbread baked on one type of rye kernels	Fructan	Rye kernels 18 samples from an experimental field, Sweden	• Fructan via enzymatic and spectrophotometry method [18] • Result presented in dry weight • Duplicate analysis
[14] Haskå et al. 2008	Two cultivars of wheat grain and five milling fractions of the wheat	Fructan	Two cultivars, one sample conventionally and organically grown. One cultivar conventionally grown in Sweden	• Enzymatic [18] • Duplicate analysis • Result presented in dry weight
[21] Yao et al. 2014	Seventy-three food items	Sorbitol and mannitol	Five grocery stores, five green grocers, Australia Melbourne	• HPLC with ELSD • Triplicate analyses • Result presented in fresh weight

HPLC High performance liquid chromatography, ELSD Evaporative light scattering detection

When a relevant FODMAP value was available it was assigned to the food item or dish in the database in Dietist XP. White bread is given as an example here: values per 100 g white bread: 0.27 g fructose [16], 1.02 g fructan [20] and 0.20 g GOS [8] were added to the database. All values assigned to the database are in fresh weight. A few values in dry weight have been converted to fresh weight using the data on water content from the food composition tables from Swedish National Food Agency (Swedish food composition tables, SFCT).

### Single ingredient food items

The majority of the food items in the database are single ingredient food such as fruits, fruit-juices, vegetables, root-vegetables, milk-products as well as couscous, rice, oatmeal, pasta, potato- chips, rice cakes (plain or flavored), and legumes. When FODMAP-content data was missing the food item was assigned with the same value as a similar food item in Dietist XP, such as fresh broccoli and frozen broccoli. Similarly, brown beans and white beans originate from same plant family as kidney beans and were assigned the values for kidney beans, and bulgur was assigned with the same value as couscous.

### Composite food items

The FODMAP values for composite foods, consisting of several FODMAP containing ingredients, were obtained through a recipe calculation procedure [22]. The ingredients list from the data sources were matched with the most similar food item in the SFCT on which the database is built. As neither the SFCT nor the articles presenting data on FODMAP content present the exact amount of ingredients the food items were matched based on quantity of content. This was true for bread in particular, see section “Fructan” below.

### Fructose

Fructose is jointly absorbed with glucose using GLUT-5 and GLUT-2 [23] transporters and fructose in excess of glucose is more likely to be malabsorbed [7]. Fructose will be fermented whenever not properly absorbed. When calculating the total FODMAP value, fructose in excess of glucose is approximated by taking the monosaccharide content from the food records, in most foods free fructose is accompanied by glucose in various proportions [7]. If the glucose content was higher than the fructose content the excess fructose value is set to 0 [24]. Swedish fructose and glucose values from an appendix from the Swedish National Food Agency [15], analyzed using GC, were used when available. When data on a food item was missing, recent data on free fructose analyzed with different HPLC techniques and fresh weight were used [8–10, 16]. A few values were taken from the Finnish food composition database Fineli [25].

### Fructans

Swedish values for food items in dry weight were chosen for whole grain rye, soft and- crispbreads as well as values for wheat flour and other milling fractions of wheat [13, 14, 26, 27]. Analytical values of fructan content of Swedish sources were transformed from dry weight to fresh weigh using the water content in food items from SFCT [16]. Values from Australia [8–10] and U.K were also used [20].

Total fructan content in a food item is measured using an enzymatic/spectrophotometric method [18]. In a few cases (< 15) when data on total fructan content in a food item from enzymatic analyses was missing, values from separate HPLC analyses of the main fructo-oligosaccharides e.g. nystose (GF2), kestose (GF3) and 1F-β- fructofuranosylnystose (GF4) were summed up to give an approximation of the total fructan value [12]. When there were discrepancies between papers regarding presence of fructan in a food item, such as bananas, the paper detecting fructan was chosen as the source of the assigned value to ensure that all fructan content in food was captured.

Breads from SFCT were grouped depending on their main type of flours e.g. white bread, granary bread, whole grain/meal wheat bread, sifted rye breads containing both rye and wheat flour, whole meal/grain rye breads and yeast fermented crisp bread with sourdough or non-leavened, non-yeast crisp bread. Content of yeast or sourdough was one of the main factors to take into account in the matching as fermentation is known to influence the fructan value [13]. A white bread in Sweden generally contains wheat flour, water, wheat gluten, yeast, with or without sourdough, and differs slightly from the Australian bread which often contains added soy flour [8]. The ingredients were matched to the description of bread type in published data and/or the company’s website to see the type of ingredients and type of flours in the product and the mean value was assigned, such as the values for gluten-free breads from Whelan [20] and Biesiekierski [8].

The majority of the analyzed Australian cereals and muesli came from large multinational companies that are sold internationally, e.g. Kellogg’s cornflakes, Weetabix, All-Bran and Rice Crispies. Other cereals and muesli was matched based on the description of the product [8], especially noting if the muesli contains dried fruit which is the main source of fructose in the food item. The same procedure was used for biscuits.

The values for composite foods containing wheat flour were calculated with 1.3 g of fructan/100 g wheat flour, the mean value from three wheat cultivars analyzed in Sweden [14].

### Lactose

Most values for lactose were derived from Swedish [28] and Finnish [29] dairy companies who have comprehensive data of lactose content in their products although a few Swedish values came from the Swedish National Food Agency.

### GOS and polyols

Australian data regarding GOS and polyols in fruits, vegetables and chewing gum [8, 10, 21] were used as no data from Europe was available. Carbohydrates separately analyzed with HPLC e.g., raffinose and stachyose were summed up to give a value of GOS. Sorbitol and mannitol were summed up and called polyols. Values for composite meals containing GOS were calculated by using information about components from food business sites. Values for cough-drops were found on business websites [30].

### Calculation of FODMAP intake from food diaries

#### Collection of food records

The present study includes 117 estimated four-day food records randomly selected from the Swedish nationwide diet survey Riksmaten [31] performed from May 2010 until July 2011. Sample size was determined based on constraining the uncertainty around the estimated mean of fructan, as fructan intake is a key parameter for FODMAP diet treatment in clinical practice, to within 10% of the expected mean value. This ensures sufficiently precise estimates of average intake. Assuming an SD of 2.5 and a 95% confidence interval, approximately 110 individuals were required. The achieved sample size slightly exceeds this requirement at 117.

Eligible participants of Riksmaten, 5000 randomly selected Swedish adults aged 19–80 years, were sent a letter with written information about the study and were phoned a few days later by a trained interviewer from Statistics Sweden and asked if they wanted to participate in the Riksmaten study. A total of 1797 persons chose to participate. Participants in the present study were selected to serve as matched healthy controls for 117 patients in another study not yet published and individuals with diabetes, kidney-disease, lactose intolerance or celiac disease had been excluded [31]. The mean age was 39 years (range 18 to 70 years), 89 were women (76%). Body Mass Index (BMI) was on average 24.8. The study Riksmaten was approved by the Regional Ethical Review board at Uppsala University (registration number: 2010/060). All participants gave oral consent.

All participants received written and oral information about keeping a food diary. Food intake was estimated using a portion size guide to facilitate the recording. Participants were instructed to record their food intake immediately after every meal and encouraged to eat as they normally do. Food intake was entered on a website belonging to the Swedish National Food Agency. If a participant was unable to use the computerized web log, a trained person at Statistics Sweden phoned the participant and recorded the food intake during the call. The food records covered all weekdays and seasons on a group level to cover weekday versus weekend and seasonal variations in food intake.

#### Calculation of FODMAP content in food records

The 117 four-day food records were entered into Dietist XP, version 3:2, with the expanded database, by a registered dietitian. Composite dishes were entered when available in the database and otherwise broken down into their ingredients. Each food record was summarized in Dietist XP and the average intake in grams per day and 95% confidence interval per FODMAP was calculated. Each FODMAP was tabulated by magnitude of most common eaten food item, see supplementary files.

### Patient and public involvement

Patients or representatives from the public were not involved in the planning or execution of this research project.

## Results

### Expanding the food database with values of FODMAP content

A total of 998 of the 2039 food items or dishes in the database were given values for FODMAP content. Finally, 62 food items that did not exist in the database but were present in the food records used to assess FODMAP intake in the general population were added to the database as new food items e.g. lactose free products, spelt flour and soy yoghurt. A total of 1805 values of FODMAP content across 1060 (as several types of FODMAPs are present in the same food item) different food items and dishes were added to the database in Dietist XP (Fig. 1), across the following categories: cereal and grain products such as flour, bread, pasta and cereals (339); vegetables, root vegetables, potatoes and legumes (193) and desserts, snacks and candy (154); fruits and berries including juices, jam and marmalade (146); dairy products including cheese, margarines and butter (139). The remaining 89 food items belong to composite food items or dishes not clearly belonging to any specific food category (Fig. 1). A total of 1350 FODMAP values (75%) were assessed from direct measurement analyses. In total 627 fructose values, 482 fructan values, 328 lactose values, 205 GOS and 163 polyol values were added to the database.

### FODMAP intake calculated from food records

The mean intake of total FODMAPs, fructose, fructan, lactose, GOS and polyols as reported in the four-day food records from the general population is presented in Table 2 together with FODMAP intake values reported in previous studies.

**Table 2:** Mean intake of FODMAPs (95% confidence interwal) in four-day food records from 117 adults from the Swedish nationwide study Riksmaten. Mean values from previous studies are included for reference

FODMAP content (g/day)	Present study	Barret 2010 Australia [32]	Halmos 2014 Australia [33]	Anderson 2015 U.K [34]
Monosaccharides	30.7 (28.6–32.8)			
Glucose	–	24.1	25.3	–
Fructose	15.2 (14.2–16.2)	19.8	19.2	–
Excess fructose^a^	0	0	0	–
Total oligo- saccharides^b^	3.89 (3.65–4.18)	3.62	3.5	–
Fructan	3.46 (3.24–3.68)	3.0	–	3.9
Galacto-oligosaccharides (GOS)	0.43 (0.39–0.47)	0.62	–	–
Lactose	14.2 (12.5–15.7)	17.7	11	–
Polyols	1.31 (1.03–1.57)	1.54	2.0	–
Total FODMAP^c^	19.4	23	16.5	–

^a^ Fructose in excess of glucose is estimated using the monosaccharide content from the food records. The fructose content is subtracted. If glucose contant is higher than the fructose value the excess fructose value is 0

^b^ Total oligosaccharides = Galacto-oligosaccharides + fructans

^c^ Total FODMAPs = excess fructose + total oligosaccharides + lactose + polyols

### FODMAPs in commonly eaten food items in Sweden

Measured in grams, the most commonly eaten food items containing fructose were beverages, fruits and vegetables. Most commonly eaten food items and dishes containing fructan were wheat, rye, fruits and vegetables. For lactose: milk and yoghurt products; GOS wheat and rye breads. The most commonly eaten polyols food items were vegetables and fruits.

The listed FODMAP values for the ten most eaten food items in fructose, fructan, lactose, GOS and polyols (mean intake of food item in gram per day) from the 117 four-day food records are presented in Supplementary Table 1. Nineteen of the 50 (38%) food items were rye and wheat- based food such as wholegrain rye bread, white bread, pizza, pasta and pancakes. On the top 50 list eight food items were fruits (16%), eight food items were vegetables (16%) and seven were meals including pizza, pasta and lasagna (Supplementary Table 1). Meals containing several different sources of FODMAPs such as flour, vegetables, dairy products and cereal products contributed to 18 food items (breads, pasta, pizza) on the top 50 list (Supplementary Table 1).

Supplementary Tables 2, 3, 4, 5, 6 list the 20 food items and their FODMAP values presented in order of magnitude describing the highest content of FODMAPs (fructose, fructan, lactose, GOS and polyols) per 100 g food item.

## Discussion

This study demonstrates that expanding an existing food nutrition database with FODMAP values based on information from the literature can be used to calculate the intake of FODMAPs from food diaries. The mean total FODMAP intake of 19 g per day in Swedish adults by the four-day food records calculated using the expanding software. Products made of rye and wheat grains, as well as fruit and vegetables were the most common food items among the most commonly eaten FODMAPs in Sweden. Wheat and rye breads contain small amounts of fructan and GOS, and potatoes have a low content of fructose (0.28/100 g), but they are frequently consumed and therefore present among the most eaten food items containing FODMAPs. Even though pulses are the richest source of GOS (0.19 g per 100 g for canned chickpeas and 1.88 g/100 g boiled split peas) [9] but as they were not frequently consumed the mean intake was less than a gram per day in the investigated population.

The mean total FODMAP intake of 19 g per day in the present study is in concordance with data from previous studies of healthy populations assessing FODMAP intake from food records (Table 2) [32–34]. These studies are comparable regarding demographics; in the present study the mean age was 39 years (range 18–79) and 76% were women which is similar to previous studies by Barret (Australia): median age of 36 years (range 23–72) 67% women; Halmos (Australia): median age 31 years (range 23–60) 75% women and Anderson (UK): mean age of 36.6, 64% women. The food intake is similar between the countries because staple food as wheat bread and pasta, dairy products, fruits and juices are eaten often, while beans and lentils as well as sugar polyols are consumed less frequently. Although the fructan intake is similar the contribution to the intake of fructan differs between the countries. For example, in Sweden soft and hard rye bread contribute to the fructan intake and in UK and Australia its more common to eat wheat bread.

Swedish values were the first-hand choice for the database and international sources were used to make the database as complete as possible. Most of the international values added to the database were from Australia but the food items analyzed were mainly from brands from larger international companies including cereals commonly eaten in Sweden such as oatmeal, All bran, Cornflakes and Weetabix [8]. In addition, fruits are often imported and of international brands such as kiwi, grapes (Thomson), pears and apples (Granny Smith, Pink Lady, Jonathan). The international food industry may contribute to the similar level of FODMAP intake between this study and previous studies on FODMAP intake from Australia and the UK.

The main difficulty with assigning FODMAP values related to different varieties of bread. Only five out of 20 rye crispbread in the Swedish National Food Agency food database are air leavened and yeast free rye crispbread similar to the rye crispbread analyzed in Australia. The most common crispbreads in Sweden are the sourdough and yeast fermented breads containing less fructan (3.20 g/100 g) than air leavened bread, this is probably due to fructan degradation during yeast fermentation [13]. Many other factors influence fructan content in wheat and rye breads; the genotype of grains, growing conditions such as greenhouse or fields [35], milling fractions [14], yeast content, fermentation or whether the breads contains sourdough [13]. The amount of added granary may also affect the fructan value [35]. Hence, a large variation in fructan content between various breads and between countries might be expected. In addition, a typical rye bread in Sweden differs from a rye bread in Australia in terms of the proportions of rye. A Swedish rye bread often contains 100% rye flour while an Australian rye bread tends to contain a proportion of wheat flour which is the equivalent of a sifted rye bread in Sweden. However, there was a good agreement when comparing the fructan content in soft breads containing a mix of rye and wheat flour from the U.K 1.09 g/100 g [20], Australia 1.07 g/100 g [8] and Sweden (mean of eight types of breads) 1.02 g/100 g [27]. There was also a good agreement regarding fructan content in wholegrain rye soft breads between Sweden (1.14 g/100 g) [14] and the U.K (1.95 g/100 g) [20], as well as in air leavened rye crispbread analyzed in Sweden (4.40 g/100 g) and Australia (4.60 g/100 g). This indicates that values from different part of the world are applicable in many countries and that the result is generalizable. Similarly, wheat flour values from Sweden 1.30 g/100 g in fresh weight [14] is in line with a study from Australia analyzing 12 samples of wheat flour with a mean fructan value of 1.14 g/ 100 g food item fresh weight [36].

### Limitations and strengths

There are some limitations that should be taken into consideration regarding the current study. The quality of the database is only as good at the quality of the analyses performed in the data sources. The samples should include seasonal variation, stage of ripeness and genetic variation and so on [37] and data on food items with FODMAP values derived from a laboratory using a systematic approach to analyze FODMAPs would have been desirable. As this is currently not feasible, we aimed to include the best available data. All sources of FODMAP values have used standardized, established analytical methods. The database consists of 1350 (75%) values from direct measurements and the rest are values for composite foods calculated from values from direct measurements and from food composition tables. In addition, the procedure for our data collection has been carefully performed. A similar approach to assign values to a database has previously been used in glycemic index studies [38]. It should be noted though that the FODMAP content in food varies depending on a multiple of factors, such as degree of ripeness [10, 11], therefore it may be a large variation in FODMAP values between samples. It has not been possible to include measures of deviation such as confidence intervals in the database as most reports of FODMAP values are reported as means only. In addition, the sample size in the present study was calculated based on fructan values based on its high clinical relevance, and the mean intake of FODMAPs that are uncommon in the diet such as polyols is determined with a confidence interval wider than 10%.

Assessment of food intake is a difficult task and main sources of errors often occur due to misreporting, which include both under-reporting and under eating. The self-reported mean energy intake (EI) was in the current study on average 8.1 MJ (1935 kcal) ± 598 kcal per day and the mean energy expenditure (EE) was 11.2 MJ (2668 kcal) ± 415 per day (based on BMR, WHO equation, and a crude questionnaire regarding the participant’s physical activity level). This resulted in an average EI: EE-ratio of 0.72 (95% CI: 0.69–0.79). This does indicate a possible presence of under-reporting in our data [39]. However, under-reporting is suggested to be variable and mostly involved “unhealthy” food items. Since FODMAPs food items generally are considered to be “healthy food” this may therefore not have been under-reported or may even have been over-reported instead [40]. Indeed, our findings are in accordance with a study performed in Sweden where rye and wheat were the main cereal source of whole grain intake [41], milk consumption were in accordance with the European Prospective Investigation into Cancer and Nutrition (EPIC) study [42] but lower than the figures from the Swedish Board of Agriculture [43], and apple and pear intake was in line with the consumption figures from Swedish Board of Agriculture [43].

The food diary data used in this study was collected by the Swedish National Food Agency and they were meticulous in their execution of the Riksmaten study [31]. First, the selection of the study population was done by using proportional allocation. Second, participants received oral as well as written information in the form of a booklet. The food intake was recorded prospectively on a dedicated website which may lower the risk for memory lapses and facilitate the identification of additional details [44]. Third, they alternated the starting day of food recording in order to capture both the day to day and seasonal variations. In addition, the use of estimated (i.e., the ingredients were not weighted) four-day food records in the present study is the recommended method for receiving information on mean food intake and allocation in a group of individuals [45]. Furthermore, the food records were registered by one dietitian using the same manual for all food records. However, total participation rate of the Riksmaten-survey was low (36%), with men and immigrants underrepresented and persons with high educational level overrepresented [31]. In addition, in the subgroup of Riksmaten used in the present study, subjects with kidney disease, celiac disease, and diabetes or lactose intolerance were excluded [46] and subjects in the present study are overall healthier than the general population. Further studies in other populations are warranted to ensure the generalizability of the most commonly eaten FODMAP containing food items.

### Clinical implications and future challenges

The list of the 50 most commonly eaten food containing FODMAPs and the 20 largest sources of each FODMAP are provided as supplements to this paper and may be used by health professionals as templates for counting FODMAPs content in composite meals, as well as being used as a guide to focus on targeting the most commonly eaten food items containing FODMAPs in dietary assessment in clinical settings. Further studies are needed to evaluate habitual intake and typical sources of FODMAPs in different groups of patients to facilitate interventions manipulating intake of FODMAPs in nutrition therapy. In the future, expanding the database with exclusively analyzed values including on composite foods will improve accuracy of FODMAP intake calculations from food records, especially as so called functional foods become increasingly common (produced by the purpose to have a physiological health benefit). For example, fructan, inulin and oligofructose are added to foods in a pure form, often made by extraction from chicory roots and used as functional food ingredient. Inulin has a neutral taste and is used as a replacement for fat in diet products and is used in dairy products, ice-cream, margarine, dressings, chocolate and cereals [47]. An additional challenge is the high content of FODMAPs in many non-food items consumed orally. Polyols is the major sweetener in sugar free cough drops and chewing gum including non-smoking products and one study showed that almost half of liquid medications contained sorbitol [48].

## Conclusion

Intake of FODMAPs as calculated using the extended database were in line with previous studies supporting its use of the database both research and in clinical interventions. Rye and wheat-based food items and dishes were identified as the most common food items containing FODMAPs consumed by adults in Sweden. The lists of the most commonly eaten FODMAP food items are provided and may be used to facilitate FODMAP diet interventions.

## Supplementary information

**Additional file 1: Supplementary Table 1.** FODMAP content of food items eaten in the largest quantity, presented as mean intake of the estimated four-day food records (*n* = 117). **Supplementary Table 2.** Lists present the 20 food items with the highest content of fructose in g per 100 g food item from the extended database. Fructose in excess of glucose is 0 if glucose content is higher than the fructose content. **Supplementary Table 3.** Lists present the 20 food items with the highest content of fructan in g per 100 g food item from the extended database. **Supplementary Table 4.** Lists present the 20 food items with the highest content of lactose in g per 100 g food item from the extended database. **Supplementary Table 5.** Lists present the 20 food items with the highest content of GOS in g per 100 g food item from the extended database. **Supplementary Table 6.** Lists present the 20 food items with the highest content of polyols in g per 100 g food item from the extended database.

## Data Availability

Access to the extended FODMAP database in Dietist XP are available from Therese Liljebo (therese.liljebo@ptj.se) upon reasonable request.

## Abbreviations

SCC: Short chain carbohydrates
GOS: Galacto-oligosaccharides
IBS: Irritable bowel syndrome
IBD: Inflammatory bowel disease
HPLC: High performance liquid chromatography
ELSD: Evaporative light scattering detection
GC: Gas- chromatography
FFQ: Food Frequency Questionnaire
European: Prospective Investigation into Cancer and Nutrition (EPIC)
EI: Energy intake
EE: Energy expenditure
