# Supplementary material for: Presence of Fermentable Oligo-, Di-, Monosaccharides, and Polyols (FODMAPs) in commonly eaten foods: extension of a database to indicate dietary FODMAP content and calculation of intake in the general population from food diary data
Source: BMC Nutr. 2020 Sep 18;6:47. doi: 10.1186/s40795-020-00374-3 (PMC7499970; doi:10.1186/s40795-020-00374-3)
Supplement: Supplementary file 1 — Additional file 1: Supplementary Table 1. FODMAP content of food items eaten in the largest quantity, presented as mean intake of the estimated four-day food records (n = 117). Supplementary Table 2. Lists present the 20 food items with the highest content of fructose in g per 100 g food item from the extended database. Fructose in excess of glucose is 0 if glucose content is higher than the fructose content. Supplementary Table 3. Lists present the 20 food items with the highest content of fructan in g per 100 g food item from the extended database. Supplementary Table 4. Lists present the 20 food items with the highest content of lactose in g per 100 g food item from the extended database. Supplementary Table 5. Lists present the 20 food items with the highest content of GOS in g per 100 g food item from the extended database. Supplementary Table 6. Lists present the 20 food items with the highest content of polyols in g per 100 g food item from the extended database. [file 40795_2020_374_MOESM1_ESM.pdf]

Supplements

Supplementary Table 1. FODMAP content of food items eaten in the largest quantity, presented as mean intake of the estimated four-day food records (n=117). Added FODMAP values for each FODMAP given in g/day with reference in parenthesis. The ten food items contributing the most to the intake of each FODMAP are listed, a few food items containing several types of FODMAPs are listed more than once.

Food items	Mean intake of food item g/day	Fructose (g/100g)	Glucose (g/100 g)	Fructose in excess of glucose (g/100 g)	Fructan (g/100g)	Lactose (g/100g)	GOS (g/100 g)	Polyol (g/100 g)	Total FODMAP content (g/100 g)	Analytical method	Comments
Fructose											
Orange juice	94	2.8 (1)	3.4 (1)	0	0	0	0	0	0	GC	
Potato	38	0.4 (2)	0.4 (2)	0	0	0	0	0	0	GC	
Banana	35	2.6 (3, 4)	4.4 (4)	0	0.5 (5)	0	0	0	0.5	Enzymes, HPLC with ELSD, ion Chromatographic	Mean value ripe and firm
Cola drinks	33	3.4 (6)	3.5 (6)	0	0	0	0	0	0		McCance & Widdowson's The composition of foods. 1994
Tomato	27	1.4 (2)	1.4 (2)	0	0.09 (4)	0	0	0	0.09	GC, HPLC with ELSD	Fructose, mean value of cherry tomato, tomato cultivated in mars, tomatoes cultivated in august. Fructan, mean of

											common and Roma tomatoes.
Apple	25	4.56 (3, 4)	1.24 (3, 4)	3.32	0.01 (5)	0	0	0.77 (4)	4.10	Enzymatic analyses and HPLC and ELSD	
Cordial fruit drink red	20	0.8 (6)	0.7 (6)	0.1	0	0	0	0	0.1		
Cucumber	18	0.8 (2)	2.33	0	0	0	0	0	0	GC	
Soft drink non-carbonized	17	0.4 (6)	0.3 (6)	0.1	0	0	0	0	0.1		Fructose value counted by raw material and recipe by THL
Whole grain ryebread 7 % fiber (Kornelle)	15	1.22 (2)	0.94 (2)	0.28	1 (7)	0	0.33 (8)	0.16 (8)	1.87	GC, Enzymatic, HPLC	Fructan, mean of bread types M to T in ref. 7
Fructan											
Banana	35	2.6 (3, 4)	4.4 (4)	0	0.5 (5)	0	0	0	0.5	Enzymes, HPLC with ELSD, ion chromatographic	Mean value ripe and firm
Wheat pasta	29	0.01 (2)	0	0	0.34 (8)	0	0	0	0.34	Enzymatic	
Tomato	27	1.4 (2)	1.4 (2)	0	0.09 (4)	0	0	0	0.09	GC, HPLC with ELSD	Mean value of common and Roma tomatoes.
Whole grain rye bread 7 % fiber (Kornelle)	15	1.22 (2)	0.94 (2)	0.28	1 (7)	0	0.33 (8)	0.16 (8)	1.87	Enzymatic, HPLC	Fructan, mean of bread types M to T in ref. 7
Orange	14	2.29 (3, 4)	3.28	0	0.03 (5)	0	0	0	0.03	Ion Chromatography	

Onion	11	1 (2)	1.95 (2)	0	6.3 (10)	0	0	0	6.3	GC, enzymatic	Onion bulb. that is most common used in Sweden
Pizza Capricciosa	8	1.03	0.93	0.1	0.33	0	0	0.29	0.72		Calculated by recipe from National Swedish Food Agency
Lasagna	8	0.05	0.1	0	0.28	2.1	0	0	2.38		Calculated by recipe from National Swedish Food Agency
Whole grain rye bread 9 % (Rallarhalvor)	8	1.22 (2)	0.94 (2)	0.28	1.14 (9)	0	0.33 (8)	0.16 (8)	1.91	GC, enzymatic, HPLC with ELSD	
Clementine	7	2.29 (3, 4)	3.28	0	0.03 (5)	0	0	0	0.03	Ion Chromatography	Values for orange
Lactose											
Medium fat milk 1.5 %	90	0	0	0	0	4.9 (10)	0	0	4.9	Chromatographic	
Sour milk fat 3%	34	0	0	0	0	3.8 (10)	0	0	3.8	Chromatographic	
Milk fat 3%	23	0	0	0	0	4.9 (10)	0	0	4.9	Chromatographic	
Skim milk fat 0.5% Enriched with vitamin A and D.	17	0	0	0	0	5 (10)	0	0	5	Chromatographic	
Plain yoghurt fat 3%	15	0	0	0	0	2.7 (10)	0	0	2.7	Chromatographic	

Fruit yoghurt 2 %	11	1.1 (2)	1.2 (2)	0	0	4.1 (10)	0	0	4.1	GC, Chromatographic	
Medium fat sour milk 1.5%	11	0	0	0	0	3.8 (10)	0	0	3.8	Chromatographic	
Low fat fruit yoghurt 0.5 %	9	1.1 (2)	1.2 (2)	0	0	2.2 (10)	0	0	2.2	GC, Chromatographic	
Lasagna	8	0.05	0.1	0	0.28	2.1	0	0	2.38		Calculated by recipe from SNFA
Mashed potatoes homemade	7	0.13	0.18	0	0	1.14	0	0	1.14		Calculated by recipe from SFNA
GOS											
Whole grain rye bread 7 % fiber (Kornelle)	15	1.22 (2)	0.94 (2)	0.28	1.14 (9)	0	0.33 (8)	0.16 (8)	1.91	GC, Enzymatic and HPLC with ELSD	
Onions	11	1.38 (2)	1.95 (2)	0	6.3 (3)	0	0	0	6.3	GC, enzymatic	
Whole grain rye bread 9% fiber (Rallarhalvor)	5	1.22 (2)	0.94 (2)	0.28	1.14 (9)	0	0.33 (8)	0.16 (8)	1.91	GC, enzymatic and HPLC with ELSD	
Pancakes homemade	5	0	0	0	0.51	2.4	0	0	2.91		Calculated by recipe from SNFA
White bread fiber 5 %	5	0.27 (2)	0	0.27	1.02 (11)	0	0.2 (8)	0	1.49	GC, enzymatic HPLC with ELSD	
Whole grain rye bread 6% fiber (Lingongrova,	4	4.44 (2)	3.42 (2)	1.02	1 (7)	0	0.33 (8)	0.16 (8)	2.61	Enzymatic and HPLC	Fructan, mean of bread types M to T in ref. 7

Skördeglädje, Gott gräddat)											
Hamburger bread	4	0.27 (2)	0	0.27	1.02 (11)	0	0.2 (8)	0	1.49		
Whole meal white bread baguette sourdough 4.4% fiber. Bake off.	4	0.27 (2)	0	0.27	0.79 (8, 11)	0	0.57 (8)	0	1.63	GC, enzymatic and HPLC with ELSD	Mean of bread whole meal sliced, pitta whole meal ref. 11 and wheat whole meal and wheat whole grain from ref. 8
Wheat whole meal bread 6% fiber	4	0.27 (2)	0	0.27	0.79 (8, 11)	0	0.57 (8)	0	1.63	GC, enzymatic and HPLC with ELSD	Mean of bread whole meal sliced, pitta whole meal ref. 11 and wheat whole meal and wheat whole grain from ref. 8
Yellow split pea soup	4	0.04			0.73	0	0.73	0	0.73		Calculated by recipe from SNFA
Polyols											
Apple	25	4.56 (3, 4))	1.24 (4)	3.32	0.01 (5)	0	0	0.77 (4)	4.1	Enzymatic and HPLC with ELSD	Five types of apples, peeled and unpeeled
Whole grain rye bread fiber 7 % (Kornelle)	15	1.22 (2)	0.94 (2)	0.28	1 (7)	0	0.33 (8)	0.16 (8)	1.87	Enzymatic and HPLC	Fructan, mean of bread types M to T in ref. 7
Pear	10	6.36 (3, 4)	2.73 (4)	3.63	0	0	0	4.1 (4)	7.73	Enzymatic and HPLC with ELSD	Two types of pear, mean ripe and firm

Pizza Capricciosa	8	1.03	0.93	0.1	0.33	0	0	0.29	0.72		Calculated by recipe from SNFA
Whole grain rye bread fiber 9 % (Rallarhalvor)	8	1.22 (2)	0.94 (2)	0.28	1.14 (9)	0	0.33 (8)	0.16 (8)	1.91	GC, enzymatic and HPLC with ELSD	
Bell peppers yellow, green, red	7	2.8 (4)	4.33 (4)	0	0	0	0	0.37 (4)	0.37	Enzymatic and HPLC with ELSD	
Avocado	6	0.15(4)	0.69 (4)	0	0	0	0	0.65 (4)	0.65	HPLC with ELSD	
Corn. canned without juice	5	0.18 (4)	0.79 (4)	0	0	0	0	0.45 (4)	0.45	HPLC with ELSD	
Whole grain rye bread fiber 6 % (Lingongrova)	4	4.44 (2)	3.42 (2)	1.02	1 (7)	0	0.33 (8)	0.16 (8)	2.61	GC, enzymatic and HPLC with ELSD	Fructan, mean of bread types M to T in ref. 7
Nectarines	4	0.62 (4)	1.49 (4)	0	0.59 (4)	0		1.01 (4)	1.6	HPLC with ELSD	

Galacto- oligosaccharides GOS
Gas-chromatography (GC)
National Institute for Health and Welfare in Finland (THL).
High performance Liquid Chromatography (HPLC)
Evaporative light scattering detection (ELSD)
Swedish National Food Agency (SNFA)

Supplementary Table 2. Lists present the 20 food items with the highest content of fructose in g per 100 g food item from the extended database. Fructose in excess of glucose is 0 if glucose content is higher than the fructose content.

**Food items**	**Fructose/100g food item**	**Glucose/100g food item**	**Excess fructose/100 g food item**	**Analytical method**	**Reference**	**Comment**
Honey	38.5	32	6.5	Chromatographic analyze	(1)	
Raisins	29	29.5	0	Chromatographic analyze	(1)	
Figs dried	26.5	30	0	Chromatographic analyze	(1)	
Dates dried	21.6	27.2	0	Chromatographic analyze	(1)	
Wafers filled biscuits	21.1	22.2	0	HPLC with ELSD	(8)	
Biscuits Singoalla type	21.1	22.2	0	HPLC with ELSD	(8)	
Filled biscuits	21.1	22.2	0	HPLC with ELSD	(8)	
Cereals gluten free with fruit	16.8	19	0	HPLC with ELSD	(8)	
Preserve, orange	15.3	16.3	0	Chromatographic analyze	(1)	
Preserve, apricot, strawberry and blackberry	14.7	12.9	1.8		(6)	Value counted from similar food item, by THL.
Prunes dried	14.6	14.4	0.2	GC	(1)	
Dried fruit and dried papaya	13	13.8	0		(6)	Value counted from similar food item, by THL.

Apple purée (light in sugar)	12.7	9	3.7	Chromatography	(1)	
Apple purée	12.7	9	3.7	Chromatographic analyze	(1)	
Sundried tomatoes	12.6	10.3	2.3	GC	(2)	
Muesli whole grain including fruit/berries/ nuts	12.6	16.1	0	HPLC with ELSD	(8)	
Lingon berry jam	11.5	11.6	0	Chromatographic analyze	(1)	
Deep fried onion	11	11.7	0		(6)	Transferred value from similar food item by THL.
Cereals whole grain, seeds and raisins	9.5	9.6	0	GC	(2)	
Fruit cocktail	9	7.4	1.6	Chromatographic analyze	(1)	

Gas-chromatography (GC)
High performance liquid chromatography (HPLC)
Evaporative light scattering detection (ELSD)
National Institute for Health and Welfare in Finland (THL)

Supplementary Table 3. Lists present the 20 food items with the highest content of fructan in g per 100 g food item from the extended database.

Food items	Fructan /100 g food item	Analytical method	Reference	Comment
Garlic	17.40	Enzymatic analysis	(3)	
Jerusalem artichoke puré and boiled	12.20	Enzymatic analysis	(3)	
Jerusalem artichoke	12.20	Enzymatic analysis	(3)	
Onion	6.30	Enzymatic analysis	(3)	Bulb
Filled biscuits, Wafers	4.61	Enzymatic analysis	(8)	
Onion powder	4.50	HPLC	(5)	Value for onion powder. Only fructo-oligosaccharides with DP2, DP3, DP4
Rye crisp bread (air-leavened), non-yeast	4.27	Enzymatic analysis	(9)	
Leek	3.80	Enzymatic analysis	(3)	Mean value leek whole and leek bulb
Wheat bran	3.70	Enzymatic analysis	(12)	
Whole meal rye and crushed rye	3.52	Enzymatic analysis	(9)	
Crisp Bread (yeast, sourdough)	3.20	Enzymatic analysis	(9)	
Müsli, fiber, Energy bars e g. Start, Allbran, Special K	2.53	Enzymatic analysis	(8)	
Whole grain Cereals	2.35	Enzymatic analysis	(8)	
Semolina	2.25	Enzymatic analysis	(12)	
Wheat germ	2.25	Enzymatic analysis	(12)	
Broken Wheat	2.16	Enzymatic analysis	(12)	
Cereals Weetabix type	2.05	Enzymatic analysis	(8)	
Bulgur	2.00	Enzymatic analysis	(8)	Value for couscous
Grahams flour	1.72	Enzymatic analysis	(12)	
Whole meal rye bread	1.14	Enzymatic analysis	(9)	

High performance liquid chromatography (HPLC)
Degree of polymerization (DP)

Supplementary Table 4. Lists present the 20 food items with the highest content of lactose in g per 100 g food item from the extended database.

Food items	Lactose /100 g food item	Analytical method	Reference	Comment
Whey cheese 49 % fat	48	HPLC	(13)	
Whey cheese butter type 5% fat	49	HPLC	(13)	Original whey cheese
Chocolate white	10	Chromatographic analyze	(1)	
Milk chocolate with and without added sugar	10	Chromatographic analyze	(1)	
Hollandaise sauce	7.70		(6)	Counted by THL on raw material of the recipe
Milk chocolate with nuts	7.7		(6)	Counted by THL on raw material of the recipe
Chocolate cream	7.50	Chromatographic analyze	(1)	
Ice cream 8,10,12 % fat	6.10	Chromatographic analyze	(1)	
Dip	5.80		(6)	Counted by THL on raw material of the recipe.
Chocolate pudding, mousse Chocolate pudding, mousse	5.70		(6)	Counted by THL on raw material of the recipe
Ice loll (cream based)	5.20		(6)	Counted by THL on raw material of the recipe.
Skim milk < 0.1% fat	5	Chromatographic analyze	(10)	
Skim milk 0.5% fat	5	Chromatographic analyze	(10)	
Gratin, stew sauce	4.9		(6)	Counted by THL on raw material of the recipe
Rice pudding (milk based)	4.9		(6)	Counted by THL on raw material of the recipe
Milk 3 %	4.9	Chromatographic analyze	(10)	

Medium fat milk 1.5%	4.9	Chromatographic analyze	(10)	
Chocolate milk drink (3 % fat)	4.9			Calculated by recipe from Swedish National Food Agency
Vanilla sauce	4.6		(6)	Counted by THL on raw material of the recipe.

High performance liquid chromatography (HPLC)
National Institute for Health and Welfare in Finland (THL)

Supplementary Table 5. Lists present the 20 food items with the highest content of GOS in g per 100 g food item from the extended database.

Food item	GOS/ 100g food item	Analytical method	Reference	Comment
Green peas	1.88	HPLC with ELSD	(8)	
Brown beans	1.44	HPLC with ELSD	(8)	Value for kidney beans, same plant family
Kidney beans, red beans	1.44	HPLC with ELSD	(8)	
White beans	1.44	HPLC with ELSD	(8)	Value for kidney beans, same plant family
Cereals whole grain 4 types (All bran type)	1.32	HPLC with ELSD	(8)	
Soy flour	0.79	HPLC with ELSD	(8)	Value for soy bean
Soy bean	0.79	HPLC with ELSD	(8)	
Vegetarian soya beef	0.79	HPLC with ELSD	(8)	Calculated by recipe using value for soy been
Miso	0.79	HPLC with ELSD	(8)	Calculated by recipe using value for soy been
Pea soup	0.73	HPLC with ELSD	(8)	Calculated by recipe using recipe from Swedish National Food Agency
Vegetarian minced soy protein	0.67	HPLC with ELSD	(8)	Calculated by recipe using value for soy been
Crisp bread wheat and rye based	0.59	HPLC with ELSD	(8)	Value for whole grain bread, wheat based bread, added rye flour and kibbled grain
Sweet almond	0.58	HPLC with ELSD	(8)	Value for LSA mix
Sunflower seeds	0.58	HPLC with ELSD	(8)	Value for LSA mix
Linseed	0.58	HPLC with ELSD	(8)	Value for LSA mix
Whole meal wheat bread fiber 6%	0.57	HPLC with ELSD	(8)	
Whole meal wheat bread fiber 13%	0.57	HPLC with ELSD	(8)	
Lentils red boiled	0.46	HPLC with ELSD	(8)	

Lentils green boiled	0.46	HPLC with ELSD	(8)
White dried beans, boiled	0.42	HPLC with ELSD	(8)

High performance liquid chromatography (HPLC)
Evaporative light scattering detection (ELSD)
Linseed, almond, sunflower mix (LSA)

Supplementary Table 6. Lists present the 20 food items with the highest content of polyols in g per 100 g food item from the extended database.

Food items	Polyols/100 g food item	Analytical method	Reference	Comment
Cough drops sugar free	97		(14)	Isomalt. Calculated by recipe
Chewing gum	41.90	HPLC with ELSD	(15)	Sorbitol
Prunes	10.80	HPLC with ELSD	(15)	Sorbitol
Dried apricot	6.0	HPLC with ELSD	(15)	Sorbitol
Blackberry fresh and frozen	4.76	HPLC with ELSD	(4)	Sorbitol
Pear	4.10	HPLC with ELSD	(4)	Sorbitol. Mean value firm and ripe
Pear nectar	3.40		(16)	Calculated using Australian analytical values for raw, unpeeled pear. (1986) Composition of Australian foods.
Cauliflower	2.96	HPLC with ELSD	(4)	Mannitol
Mushrooms	2.74	HPLC with ELSD	(4)	Sorbitol and mannitol
Plum	2.40	HPLC with ELSD	(15)	Sorbitol
Apples dried	1.90	HPLC with ELSD	(15)	Sorbitol
Celery	1.50	HPLC with ELSD	(15)	Mannitol
Apricot	1.20	HPLC with ELSD	(15)	Sorbitol
Canned apricot	1.20	HPLC with ELSD	(15)	Sorbitol. Value for apricot
Crepes with mushroom	1.20			Calculated by recipe from Swedish National Food Agency
Snow peas	1.16	HPLC with ELSD	(4)	Mannitol
Peach	1.03	HPLC with ELSD	(4)	Sorbitol and Mannitol. Mean of tree kinds of peaches
Nectarine	1.01	HPLC with ELSD	(4)	Sorbitol
Cherries canned in syrup	1		(16)	Sorbitol<br>These data were derived using a recipe approach
Muesli gluten free	0.89	HPLC with ELSD	(8)	Sorbitol

High Performance Liquid Chromatography (HPLC)
Evaporative Light Scattering Detection (ELSD)

References

1. Carbohydrate table: Swedish National Food Agency 1996.
2. Swedish National Food Agency; Available from: http://www.slv.se/
3. Muir JG, Shepherd SJ, Rosella O, Rose R, Barrett JS, Gibson PR. Fructan and free fructose content of common Australian vegetables and fruit. Journal of Agricultural and Food Chemistry. [Research Support, Non-U.S. Gov't]. 2007 Aug 8;55(16):6619-27.
4. Muir JG, Rose R, Rosella O, Liels K, Barrett JS, Shepherd SJ, et al. Measurement of short-chain carbohydrates in common Australian vegetables and fruits by high-performance liquid chromatography (HPLC). Journal of Agricultural and Food Chemistry. [Evaluation Studies Research Support, Non-U.S. Gov't]. 2009 Jan 28;57(2):554-65.
5. Campbell JM, Bauer LL, Fahey GC, Hogarth AJCL, Wolf BW, Hunter DE. Selected Fructooligosaccharide (1-Kestose, Nystose, and 1F-β-Fructofuranosylnystose) Composition of Foods and Feeds. Journal of Agricultural and Food Chemistry 1997;45(8):3076-82.
6. Available from: http://www.fineli.fi
7. Rakha A, Åman, P, Andersson, R. Characterisation of dietary fibre components in rye products. Food chem. 2009;119:859-67.
8. Biesiekierski JR, Rosella O, Rose R, Liels K, Barrett JS, Shepherd SJ, et al. Quantification of fructans, galacto-oligosacharides and other short-chain carbohydrates in processed grains and cereals. J Hum Nutr Diet. 2011 Apr;24(2):154-76.
9. Andersson R, Fransson G, Tietjen M, Åman P. Content and Molecular-Weight Distribution of Dietary Fiber Components in Whole-Grain Rye Flour and Bread. Journal of Agricultural and Food Chemistry. 2009 2009/03/11;57(5):2004-8.
10. ARLA. [2015-09-15]; Available from: http://www.arla.se/
11. Whelan K, Abrahmsohn O, David GJ, Staudacher H, Irving P, Lomer MC, et al. Fructan content of commonly consumed wheat, rye and gluten-free breads. Int J Food Sci Nutr. [Research Support, Non-U.S. Gov't]. 2011 Aug;62(5):498-503.
12. Haskå L, Nyman M, Andersson R. Distribution and Characterisation of fructan in wheat milling fractions. Journal of Cereal Science. 2008;48:768-74.
13. Fjällbrynt. [2015-09-15]; Available from: http://www.fjallbrynt.se/produkter/messmor-natur/
14. CLOETTA. [2015-09-15]; Available from: http://www.cloetta.se/varumarken-och-produkter/lakerol/
15. Yao CK, Tan HL, van Langenberg DR, Barrett JS, Rose R, Liels K, et al. Dietary sorbitol and mannitol: food content and distinct absorption patterns between healthy individuals and patients with irritable bowel syndrome. J Hum Nutr Diet. [Randomized Controlled Trial]. 2014 Apr;27 Suppl 2:263-75.
16. NUTTAB. Available from: http://www.foodstandards.gov.au/consumerinformation/nuttab2010/
